# On the Question of the Advisability of Ligaturing the Jugular Vein in the Treatment of Sigmoid-Sinus Thrombosis

**Published:** 1901-03

**Authors:** J. Lacy Firth

**Affiliations:** Assistant-Surgeon to the Bristol General Hospital.


### ON THE QUESTION OF THE ADVISABILITY OF LIGATURING THE JUGULAR VEIN IN THE TREATMENT OF SIGMOID-SINUS THROMBOSIS.

BY

J. Lacy Firth, M.S., M.D. Lond., F.R.C.S. Eng.,

*Assistant-Surgeon to the Bristol General Hospital.*

---

Many cases of thrombosis of the sigmoid sinus, secondary to inflammation of the middle ear, have been successfully treated without ligature of the internal jugular vein. Some cases, indeed, have terminated favourably although neither the sinus itself nor the jugular vein have been directly treated. I have myself, in the practice of other surgeons, seen one case in which pyæmic abscesses formed in the sterno-clavicular joint and over the olecranon, and another in which there were signs and symptoms of lung implication secondary to otitis media, and yet these cases recovered after antrectomy alone, the sinuses and jugular veins not being touched. In both of them there is great probability that the sinuses were at least partially thrombosed, and that if they had been obliterated by operation early in the course of the disease, with or without ligature of the jugular vein, the pyæmic developments might have been prevented; but obviously these procedures were not necessary to save life.[1] The cases are mentioned, not to show that antrectomy alone is ever the proper treatment in suspected cases of sinus thrombosis, but rather to show that, though recovery ensued, the treatment adopted was hazardous. In many cases of this disease, however, the thrombosed sinuses have been cleared of their septic contents without the corresponding jugular veins being ligatured, and yet the subsequent course of the cases has apparently been as satisfactory and as free from hazard as in other cases where the sinus and vein have both been dealt with.

[1] These cases may have been examples of aural pyæmia without sinus thrombosis—a form of pyæmia which is sometimes, though rarely met with.

The routine treatment adopted by many surgeons in cases where thrombosis of the sigmoid sinus is suspected to exist is: firstly, to expose and disinfect the mastoid antrum and cells, and the tympanum; then to expose the sinus and settle the question as to whether it is or is not thrombosed; then, if the sinus is thrombosed, to expose the jugular vein and obliterate its channel, either by ligaturing the vein in continuity, or by dividing it after doubly ligaturing, or by excising it from near its origin at the base of the skull to near its termination in the subclavian vein close to the clavicle; and finally to freely lay open the thrombosed sinus and remove the thrombus, and thoroughly disinfect the vessel. If the jugular vein has to be exposed, it would in most cases be the best to make that the first step in the operation, as the neck wound would then have a better chance of escaping infection from contaminated hands or instruments. In some cases, however, the upper part of the jugular vein will be surrounded by tissue already infected, and in such cases there would be no great advantage in making the ligature of the vein the first step in the operation. But the diagnosis of thrombosis of the sigmoid sinus in the early stages, when operation is most likely to be beneficial, is usually problematical, and the sinus should be exposed before the vein is ligatured lest the diagnosis prove incorrect. In any cases, however, in which the ligature is used, it should be applied before the thrombotic material which the sinus contains is disturbed, for obvious reasons. The excision or division of the internal jugular vein bars at once the path by which septic emboli, breaking away from a disintegrating clot in the sigmoid sinus, most readily pass to the lungs and other organs, though it does not bar the only path by which such emboli travel, for it leaves open in many cases the anterior and posterior condylar and stylo-mastoid veins, and also the lateral sinus itself may convey the particles in its reversed current to the opposite jugular vein. If the jugular vein itself has become infected, and contains disintegrating thrombus, the wisdom of applying a ligature below the thrombus admits of no question. But the fact that many cases of sinus-thrombosis have recovered satisfactorily without ligature of the vein raises the practical question—Are there any signs or symptoms by which the surgeon called upon to treat these cases can decide, either when operating or before, whether it is safe or unsafe to leave the jugular vein untouched? From the pathological point of view it is obvious that nothing will be gained by ligaturing the jugular—firstly, if every particle of septic thrombotic material likely to pass from the sinus to the vein has been or can be removed through an opening made into the sinus itself; and secondly, if the lumen of the jugular vein is blocked by firm clots, which can act like a ligature, if that clot can be saved from disintegration by removing the septic material lying in the sinus above it as just described.

This question of the advisability of tying the jugular as a routine step in the treatment of sigmoid-sinus thrombosis can only be settled by a careful study of recorded cases. It is a very important question to settle, for the operation of antrectomy and mastoidectomy, coupled with that necessary to expose and obliterate the sigmoid sinus, is often sufficiently long to tax severely the patient's vital powers without the addition of another operation on the neck. Moreover, it is surgically unsound to perform any operation that does not offer advantages to the patient commensurate with the risks from the shock, hemorrhage, anæsthesia, and chances of septic infection which may attend the operation. Unfortunately it will always be in the cases of sinus thrombosis of the severest type and the longest standing, those in which the patient is least able to bear a prolonged operation, that ligature of the jugular vein will be most essential for successful treatment. It is with the object of adding a fractional contribution to the data for the study of this important question that I record the following case:—

J. W., a male, aged 26, was admitted to the Bristol General Hospital on June 4th, 1900, and gave the following history. Six weeks before his present illness began he had discharge from his right ear, lasting for about a fortnight. There had been no pain or vomiting with this. A period of three or four weeks followed, during which he had no otorrhœa and felt quite well. On two previous occasions he had had ear discharge. Six days before admission, his present illness set in with malaise, vomiting, and a throbbing pain in the right ear, temples, and forehead. These symptoms had gradually become more severe, and he had had four shivering fits, the last occurring in the hospital and his temperature with it rising to 103°. The other shivering attacks were on the the first, second, and third days of his illness. He had become very prostrate. Vomiting did not recur in the hospital. I saw him first on June 5th. There was a tender gland palpable just below the apex of the mastoid process on the right side, of elongated shape, and not larger than a cherry-stone. No tenderness or cord-like thickening was found along the course of the jugular vein. The median frontal vein, and some veins over the base of the mastoid, were rather full, but none of them felt cord-like, and nothing abnormal could be felt in the sub-occipital triangle. There was no mastoid tenderness whatever, and no ear discharge. A watch could only be heard in contact on the affected side, but on the other side could be heard well from a considerable distance. His intelligence was normal. The eyes were normal in all respects. After well syringing the right ear, and washing away a black mass and whitish flakes, the speculum showed nothing recognizable as tympanic membrane. The ossicles could not be seen. Some rhonchi were heard in the chest and a faint systolic apical heart bruit. The pulse was 98. The headache was said to be very severe, and obviously the patient was very prostrate.

*Operation.*—This was performed on the seventh day of illness, and about two hours after I first saw the patient. A curved incision was made half an inch behind the insertion of the pinna, and the mastoid antrum opened with hand-gouges. The bone was hard and the antral cavity deep, so that a long time was taken in exposing it. Minute beads of pus were twice seen before the antrum was reached, but nothing came from that cavity until a small spoon was introduced, which brought away offensive caseous-looking material. After well scraping out the antrum, and the tympanum also, and syringing through several times, these two cavities were plugged with iodoform gauze. A horizontal incision was carried backwards, and a disc of bone removed over the sigmoid sinus an inch behind the meatus. Watery flocculent pus immediately escaped from an extra-dural collection lying in the sigmoid groove and over the posterior surface of the petrous bone. The sinus was swollen and yellowish in colour, the outer wall looking sloughy. It was further exposed in both directions with bone forceps and then laid open along the whole length exposed, and was found to contain blood-clot, which was dark coloured externally, but greyish in its central part, where it appeared to be disintegrating. The clot was removed with a sharp spoon. It cannot have been more than one and a half inches long, as shown by the short distance it was necessary to pass the spoon to excite free bleeding in both the jugular and torcular directions. The hemorrhage was easily stopped by plugging the sinus and wound with iodoform gauze. The temperature on the following day was normal. The plugs were changed in 48 hours and hemorrhage occurred during their removal, which was again easily arrested. Then for eight or nine days good progress was made, the patient gaining strength rapidly and feeling well. Then a swelling gradually formed under the uppermost part of the sterno-mastoid muscle, projecting forward towards the angle of the jaw, and coincidently the temperature became again elevated. The skin at this time had become eczematous around the wound. A week later the temperature again became normal, and the swelling quickly subsided. As it subsided there was very free discharge of pus from the opening in the skull, and pressure on the swelling was several times observed to cause an escape of pus from that opening. It is obvious, therefore, that the swelling was due to a collection of pus which communicated with the cranial cavity, probably through an aperture the result ot caries of the mastoid process on its deep surface, or of caries very near that process. It seemed, in fact, an abscess of the type described by Bezold, and it is remarkable that it healed up so quickly and satisfactorily without further operative treatment. From this time the progress of the patient was uneventful. I last saw him in October. The wound behind the pinna was soundly healed, and there was no ear discharge, and fair hearing on the affected side.

The successful issue of this case was no doubt due to the thorough removal of the septic thrombus in the sinus, and to the obliteration of the sinus with the gauze plugs preventing the further circulation of blood through the contaminated portion.

The effective removal of the thrombus through an incision in the sinus wall was easy, because the operation was performed before the sinus became extensively involved.

As bearing upon the question of ligature of the jugular, reference may be made to the following cases which have been recorded recently: A. R. Baker[1] opened a thrombosed sinus and curetted upwards and downwards until there was a free flow of blood each way, and tamponed with iodoform gauze. Uncomplicated recovery followed. Arthur Cheatle,[2] in treating a case, made the sinus bleed from the torcular, but not from the jugular, end. The clot was firm and dark, except at one infected spot, and for that reason the jugular vein was not tied. There was a rigor one hour after the operation, but after that progress was uninterrupted. Cheatle, commenting upon the case, says: "It seems that if healthy-looking clot can be seen well below the breaking-down area ligation of the vein is not necessary; but in order that such a condition may be found it must be well recognised that *one* rigor demands instant operation." He points out that one rigor does not necessarily mean that there is septic thrombosis of the sinus, and mentions a case of Urban Pritchard's where a severe rigor seemed to be due to suppuration in the antrum alone. But one rigor points strongly to the advisability of examining the sinus. Dundas Grant[3] reports another case. The patient was almost moribund when she arrived at the hospital. She had had repeated shiverings for a fortnight. A quantity of fœtid pus was found to be escaping

[1] *Laryngoscope*, 1900, viii. 68. [2] *Lancet*, 1900, i. 96.

[3] *J. Laryngol.*, 1900, xv. 143.

from a hole in the wall of the sinus. Broken-down clot was scraped from the sinus, but normal-looking blood-clot was found beyond this disintegrating clot in both directions, and this was left in the hope that the original cause of infection having been removed, no further infection would occur, and that the firm clot which had formed would act in the same way as a ligature. M'Kernon[1] of New York has recently recorded seven cases of sigmoid-sinus thrombosis, of which six were successfully treated, in four of which the jugular vein was not tied. All the sinuses in his cases contained pus and breaking-down clot, though in varying quantity. In the four cases in which no ligature was used the sinus was made to bleed from both ends by removing the thrombi. In the two cases in which the jugular vein was excised he appears to have been induced to operate on the vein by the fact that he could not remove clot from the lower end of the sinus and the jugular bulb sufficiently completely to excite free hemorrhage there. Yet in his comment upon treatment at the end of the paper he says: "If, on the other hand, upon opening the sinus, we find a disintegrated clot or pus, or both present, then I believe, without further manipulation above, we should, as rapidly as is consistent with carefulness, expose, ligate at the clavicle, and resect and remove the internal jugular vein of that side, to its commencement at the bulb." Macewen, in his book on *Pyogenic Diseases of the Brain and Spinal Cord*, 1893, p. 311, writes: "In the majority of instances the obliteration of the upper two-thirds of the sigmoid sinus is all that is necessary for the prevention of systemic infection."

It appears to me that a study of the records I have quoted goes far to show that ligature of the jugular vein in sigmoid sinus thrombosis need not, in many cases, be regarded as an essential part of the operation, and that it will usually be better practice not to ligate the vein in cases operated upon before embolic abscesses have formed, if a free flow of blood can be established from both ends of the sinus by removal of the thrombi, or if firm healthy-looking clot is found plugging the sinus at both ends, or at the lower end.

[1] *Laryngoscope*, 1900, viii. 294, 333.

